# Tenofovir Alafenamide for HIV Prevention: Review of the Proceedings from the Gates Foundation Long-Acting TAF Product Development Meeting

**DOI:** 10.1089/aid.2021.0028

**Published:** 2021-06-01

**Authors:** Joseph W. Romano, Marc M. Baum, Zach R. Demkovich, Frank Diana, Charles Dobard, Paul L. Feldman, J. Gerardo Garcia-Lerma, Alessandro Grattoni, Manjula Gunawardana, Duy-Khiet Ho, Thomas J. Hope, Ivana Massud, Mark Milad, John A. Moss, Fernanda P. Pons-Faudoa, Shane Roller, Ariane van der Straten, Selvi Srinivasan, Ronald S. Veazey, Doris Zane

**Affiliations:** ^1^ NWJ Group, LLC., Wayne, Pennsylvania, USA.; ^2^ Department of Chemistry, Oak Crest Institute of Science, Monrovia, California, USA.; ^3^ RTI International, Durham, North Carolina, USA.; ^4^ FJD-CMC Consulting, LLC., Ocean City, New Jersey, USA.; ^5^ Division of HIV/AIDS Prevention, National Center for HIV/AIDS, Viral Hepatitis, STD, and TB Prevention, Centers for Disease Control and Prevention, Atlanta, Georgia, USA.; ^6^ Intarcia Therapeutics, Inc., Research Triangle Park, North Carolina, USA.; ^7^ Department of Nanomedicine, Houston Methodist Research Institute, Houston, Texas, USA.; ^8^ Department of Bioengineering, University of Washington, Seattle, Washington, USA.; ^9^ Department of Cell and Developmental Biology, Northwestern University, Chicago, Illinois, USA.; ^10^ Milad Pharmaceutical Consulting, Plymouth, Michigan, USA.; ^11^ Women's Global Health Imperative, RTI International, Berkeley, California, USA.; ^12^ Department of Medicine, Center for AIDS Prevention Study (CAPS), UCSF, San Francisco, California, USA.; ^13^ Department of Pathology and Laboratory Medicine, Tulane University School of Medicine, New Orleans, Louisiana, USA.; ^14^ Intarcia Therapeutics, Inc., Heyward, California, USA.

**Keywords:** long-acting, TAF, HIV PrEP

## Abstract

The ability to successfully develop a safe and effective vaccine for the prevention of HIV infection has proven challenging. Consequently, alternative approaches to HIV infection prevention have been pursued, and there have been a number of successes with differing levels of efficacy. At present, only two oral preexposure prophylaxis (PrEP) products are available, Truvada and Descovy. Descovy is a newer product not yet indicated in individuals at risk of HIV-1 infection from receptive vaginal sex, because it still needs to be evaluated in this population. A topical dapivirine vaginal ring is currently under regulatory review, and a long-acting (LA) injectable cabotegravir product shows strong promise. Although demonstrably effective, daily oral PrEP presents adherence challenges for many users, particularly adolescent girls and young women, key target populations. This limitation has triggered development efforts in LA HIV prevention options. This article reviews efforts supported by the Bill & Melinda Gates Foundation, as well as similar work by other groups, to identify and develop optimal LA HIV prevention products. Specifically, this article is a summary review of a meeting convened by the foundation in early 2020 that focused on the development of LA products designed for extended delivery of tenofovir alafenamide (TAF) for HIV prevention. The review broadly serves as technical guidance for preclinical development of LA HIV prevention products. The meeting examined the technical feasibility of multiple delivery technologies, *in vivo* pharmacokinetics, and safety of subcutaneous (SC) delivery of TAF in animal models. Ultimately, the foundation concluded that there are technologies available for long-term delivery of TAF. However, because of potentially limited efficacy and possible toxicity issues with SC delivery, the foundation will not continue investing in the development of LA, SC delivery of TAF products for HIV prevention.

## Introduction

More than 4 years ago, the Bill & Melinda Gates Foundation (the foundation) initiated investments designed to develop long-acting (LA) delivery of antiretroviral (ARV) drugs for the prevention of HIV transmission in both men and women. This strategy was driven by the challenges of required daily adherence to an oral pill regimen like Truvada [FTC/tenofovir disoproxil fumarate (TDF)] for oral preexposure prophylaxis (PrEP),^1,2^ particularly for adolescent girls and young women (AGYW). There were two top line requirements necessary to achieve this goal: (1) identification of an appropriate active pharmaceutical ingredient (API), and (2) a compatible LA delivery technology that could achieve safe and effective levels of the priority API. As will be explained hereunder, tenofovir alafenamide (TAF) became the priority API at the foundation for LA HIV prevention. These new foundation-supported efforts focusing on TAF were conducted independently and in parallel with development of other LA prevention products, also supported in partnerships with the foundation. These included, the injectable suspension formulation of the integrase inhibitor, cabotegravir^3^ (CAB-LA; GlaxoSmith-Kline; ViiV), that had originally been developed as an LA treatment option to be used in combination with the LA injectable suspension formulation of rilpivirine (Janssen)^4^; and, the 30-day dapivirine vaginal ring (International Partnership of Microbicides).^5,6^

These products were in advanced development at the time the foundation initiated its investments in the LA ARV portfolio described here. Of importance, both the injectable CAB and the dapivirine vaginal ring provide useful advantages for HIV prevention. Consequently, the goal of this foundation initiative was to determine if there were other LA drug-device strategies that had additional or alternative advantages, and could provide end users, particularly AGYW, with more choices.

In January 2020, the foundation convened a meeting of its active LA TAF product development grantees, along with additional experts in the field who were independently working on similar projects (see Table 1 for participants). Through the course of this meeting, data were provided by the individual foundation grantees on their specific product development efforts, including preclinical safety and pharmacokinetic (PK) findings, as well as similar product development summaries from independent groups. This article is a summary of that meeting. Owing to observations reported by several groups regarding preclinical findings of local toxicity with subcutaneous (SC) delivery of TAF, as well as potentially low efficacy as observed in nonhuman primate (NHP) oral dosing studies,^7–9^ the foundation concluded that continued investment in LA TAF products for HIV prevention was unjustified. This article summarizes the product preclinical development studies along with the data analyses conducted by the meeting participants that led to this conclusion.

**Table 1.** Groups Participating in the Long-Acting Tenofovir Alafenamide Meeting, January 2020

Group	Activity focus	Funding source
CDC	Oral dosing in NHP models of TAF and F/TAF for safety. PK and HIV prevention efficacy	CDC intramural funding, USAID
Northwestern University	LA TAF formulation development and performance evaluation; end-user acceptability studies	NIH/NIAID
Oak Crest	Nonbiodegradable reservoir implant	NIH/NIAID
Methodist Hospital	Nondegradable, refillable reservoir implant	NIAID, NIGM, Gilead Sciences
Gilead Sciences	Development of TAF/FTAF for HIV treatment and HIV prevention	Self-funded with multiple partner organizations
Intarcia	Proprietary nondegradable, osmotic pump reservoir implant	Foundation^a^
RTI International	Biodegradable implant device	Foundation^a^/USAID
University of Washington	Drugamer technology for LA injection delivery	Foundation^a^

^a^ The Bill & Melinda Gates Foundation.

CDC, Centers for Disease Control and Prevention; FTAF, FTC/TAF; LA, long acting; NHP, nonhuman primate; NIAID, National Institute of Allergy and Infectious Diseases; NIGM, National Institute of General Medicine; NIH, National Institutes of Health; PK, pharmacokinetic; TAF, tenofovir alafenamide; USAID, United States Agency for International Development.

## Background

*Why LA PrEP?* The optimal way to control a pandemic is to prevent infections with an effective, durable, safe, and acceptable vaccine that is made available to and successfully used by at-risk populations on a large scale. Unfortunately, the nature of HIV has made vaccine discovery and development extraordinarily challenging. Of importance, alternatives to vaccine strategies for HIV PrEP have also been investigated and successfully developed. Although HIV treatment has proven to be very effective in preventing HIV transmission,^10^ implementing this approach involves challenges, particularly in resource-limited settings, including availability of ARV drugs, testing capacity, clinical follow-up, and cost.

Another effective option for the prevention of HIV infection is oral PrEP. HIV oral PrEP efficacy was first demonstrated with the use of oral truvada (FTC/TDF) in men who have sex with men (MSM) and transgender women (TGW),^11^ and in sero-discordant couples.^12^ However, consistent adherence to daily oral PrEP has been challenging for many end users over a prolonged period of time, even in the context of controlled clinical trials.^1,2^ Similar adherence issues have been identified with end-user-controlled vaginal microbicides.^13^

It has been shown that an effective option for addressing the end-user adherence challenge is the use of a LA injectable ARV. Results were recently reported from the HPTN083 trial conducted in MSM and TGW. This was a double-blind, double-dummy, noninferiority trial of CAB-LA versus FTC/TDF oral PrEP.^14^ CAB-LA was shown to be three times more effective than oral Truvada in preventing HIV infection. Similar results were recently reported from the HPTN084 trial conducted in women.^15^ The apparent benefit of injectable and possibly implantable prevention products, regarding end-user adherence is supported by a number of user preference studies that have demonstrated that end users, particularly women, prefer LA product options for prevention.^16–18^

Despite the compelling results of the HPTN083 and HPTN084 trials and the supporting data from the end-user studies, there is still a need for additional LA products for HIV prevention. Because of the potency of most ARV drugs currently available for PrEP, high doses and drug loads are likely required for LA prevention, which could present feasibility challenges with conventional LA delivery technologies (injectables, implants, etc.). Another potential issue to address with LA injectable ARV products and degradable implants includes the need to mitigate possible safety risks with the potential inability to remove such products once administered.

## Strategy

In light of the potential benefits and challenges associated with current LA HIV prevention products, the foundation initiated an effort to develop new LA prevention products in 2015. This effort involved an assessment of API options for potency and compatibility with novel delivery technologies, drug release/duration potential, toxicity/safety, and PK evaluations.

### API selection

The key elements to identifying a suitable API candidate for LA prevention products were as follows: potency; safety; compatibility with potential LA delivery technologies (physicochemical properties); efficacy potential; availability for development, and, if at all possible, regulatory approval for treatment already established (note: the foundation was not pursuing new molecular entities at that time). After an internal survey and evaluation of API options that included nucleoside reverse transcriptase inhibitors [e.g., tenofovir (TFV), TAF], non-nucleoside reverse transcriptase inhibitors (e.g., rilpivirine), integrase inhibitors (e.g., CAB), and a limited number of specific earlier stage compounds that possibly met the selection criteria, TAF (Gilead)^19^ was selected. The selection of TAF was based on the potential for long duration of effect and its relatively higher potency to support its SC delivery with LA technologies.

During initiation of the project, several delivery technologies were screened for the probability of success with TAF. LA prevention products require high potency drugs because it is difficult to formulate and inject large amounts of drug under the skin or into muscle, and typical implants have limited drug-loading capacities. For example, Gunawardana *et al.*^20^ estimated that a 1-year TAF implant would need to release 51 mg of TAF (0.14 mg/day for 365 days). However, this estimate was based on scaling the active metabolite, tenofovir diphosphate (TFV-DP) PK from dog to human and targeting the estimated EC_90_ for TFV-DP in peripheral blood mononuclear cells (PBMCs) at ∼40 fmol TFV-DP/10^6^ in lysed PBMC. Other studies^19–21^ indicated a more conservative daily release rate for efficacy (e.g., 0.4–1 mg/day release), or durations of release (e.g., at least 6 months for an injectable or biodegradable implant) with the amount of drug dosed dependent on the targeted duration of effectiveness.

For example, drug needs for a 6-month duration injection or biodegradable implant can be calculated as follows: 180 days × (0.4–1 mg/day) = 72–180 mg total; and, a target duration of release of 12 months for a more durable implant: 360 days × (0.4–1 mg/day) = 144–360 mg total. Thus, it was important as part of the preclinical development programs to assess the safety of SC delivery of TAF across a wide range of exposures, as well as to gain further insights into TAF's efficacy for HIV PrEP using preclinical models to enable data-driven decisions for the progression of TAF in LA technologies into clinical studies.

### LA target product profile

Key elements of the TPP were defined as a means of identifying potential delivery technologies (injectables or implants) and assessing development feasibility and progress. These elements are summarized in Table 2.

**Table 2.** Key Target Product Profile Criteria for Candidate Products

Dosage form	SC or IM injection
	SC implant
Dose volume (injectable):	2–4 mL
Size (conventional implant):	∼4 cm long; outer diameter ∼2 mm
Alternative implant design:	Consistent with end user acceptability
Time to sustainable effective exposure:	24 h
Other key specifications/attributes:	Minimal drug burst after dosing
	No prolonged PK tail
	Scalable manufacturing
	Cost effective relative to existing PrEP products
	Administration/use consistent with comparable contraceptive products

IM, intramuscular; PrEP, preexposure prophylaxis; SC, subcutaneous.

### Candidate technologies and development partners

After a lengthy survey effort to identify potential LA TAF delivery technologies, the foundation identified the following partner grantees:

(1) University of Washington Department of Bioengineering: Injectable “Drugamer” Technology,^22^ Principle Investigator: Dr. Patrick S. Stayton
(2) RTI International: Tunable, biodegradable reservoir implant device.^23^ Principle Investigator: Dr. Ariane van der Straten
(3) Intarcia Therapeutics Inc.: Osmotic mini-pump, SC titanium implant.^24^ Principle Investigator: Dr. Paul L. Feldman

These three partners all used different technologies to achieve LA SC delivery of TAF: injection, degradable implant, and nondegradable implant. In addition to the efforts of these partners, other groups who were working independently of the foundation developing LA TAF delivery products with other sources of funding were also invited to the 2020 LA TAF Meeting. The purpose of this meeting was to review the current status of LA TAF product feasibility, and determine if any specific additional investment in these efforts was warranted. Those additional partner groups and technologies include the following:

(1) Northwestern University (NW), Departments of Biomedical Engineering; and, Cell and Developmental Biology: tunable, LA, SC reservoir implant^25^ developed in the sustained long-acting protection from HIV (SLAP-HIV) program. Principle investigators: Dr. Thomas J. Hope and Dr. Patrick Kiser
2. Houston Methodist Research Institute, Department of Nanomedicine: Transcutaneous refillable nanofluidic implant.^26^ Principle investigator: Dr. Alessandro Grattoni
3. Oak Crest Institute of Science: Nondegradable reservoir implant.^20^ Principle investigators: Dr. Marc Baum and Dr. John Moss

## Technology and Performance Summaries of the LA TAF Assets: PK and Safety

### University of Washington: drug-polymer conjugate technology

This “drugamer” technology involves conjugating a TAF molecule through a linker to a monomer, which is then polymerized into alternative final architectures (homopolymer, di-block and hyperbranched polymers, and polymer micelle configurations) using reversible addition fragmentation transfer, a controlled form of free radical polymerization.^27^ The alternative configurations generated by this process provide different levels of control over drug release from the polymer. This polymer serves as an injectable SC hydrogel reservoir, where TAF is released through hydrolysis or enzyme-mediated cleavage at the linker. This technology allows for relatively high drug loads, and the depot is held together by the hydrophobicity of the polymer formulation. The hydrophobicity and molecular architecture of the polymer formulation also helps keep the TAF drug substance stable inside this SC hydrogel depot, which is important because TAF is susceptible to degradation in aqueous environments.^28^

**FIG. 1.**
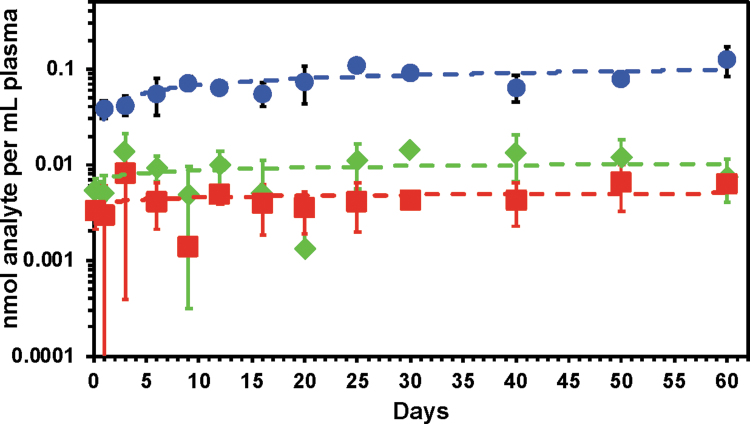
The plasma PKs were characterized for the homopolymer drugamer depots with a benzyl carbamate linker, p(Benzyl-TAFMA) with 54.5 drug wt% TAF, or an alkyl carbamate linker, p(Alkyl-TAFMA) with 73 drug wt% TAF. The polymers were formulated in DMSO at the polymer concentration of 625 and 465 mg/mL, respectively. The final dosing volume was 20 μL that corresponded to the 6.77 mg TAF/mouse. Plasma was collected at designated time points (4 h, 1, 3, 6, 9, 12, 16, 20, 25, 30, 40, 50, and 60 days). LC-MS/MS measurements of TFV and TAF were performed with isotope-labeled standards. All animal procedures and handling were performed with the approval of the Institutional Animal Care and Use Committee and kept in accordance with federal and state policies on animal research at the University of Washington. Female BALB/cJ mice, aged 6–8 weeks at time of experiments, were obtained from Jackson Laboratory (Bar Harbor, ME). *n* = 3 mice per data points. *Blue circle*: plasma TFV from the p(benzyl-TAFMA), *green diamond*: plasma TFV from p(alkyl-TAFMA), and *red square*: plasma TAF from p(alkyl-TAFMA). No TAF was measurable in the p(benzyl-TAFMA) depot. *Dash lines* are logarithmic trendlines.^29^ DMSO, dimethyl sulfoxide; LC-MS, liquid chromatography-mass spectrometry; TAF, tenofovir alafenamide; TFV, tenofovir.

Alternative versions of these formulations were evaluated in mouse PK studies (e.g., Fig. 1).^29^ No formal safety assessments were conducted in this program with this delivery system. The goal was to identify formulations for future evaluation in the dog model for PK and safety. The group reported *in vivo* PK results from mice using alternative alkyl linkers in the TAF drugamer formulations. The homopoly TAF alkyl linker formulation demonstrated consistent delivery in the mouse study for 60 days (∼0.005 nM TAF/mL plasma for a total of 6.77 mg TAF per mouse over the 60-day study). This was the first time TAF levels in plasma *in vivo* could be reported using this technology.

### RTI: biodegradable polycaprolactone TAF implant

The technology used by RTI involves the fabrication of a cylindrical implant through hot melt extrusion of polycaprolactone (PCL). The cylinder is sealed at one end and then loaded with TAF and any additional necessary excipients (e.g., polyethylene glycol; sesame, castor, or alternative oils; buffering agents) as needed. Candidate SC implants were evaluated for *in vitro* release and stability and selected for *in vivo* evaluation.

The RTI group reported a number of advances with their technology development. For example, they reported API compatibility and successful delivery of a number of different drugs besides TAF. They were also successful in adapting their implant so that it can be inserted *in vivo* with an existing trocar device, the Sino II contraceptive implant trocar (Shanghai Dahua Pharmaceuticals Co. Ltd). This implant also demonstrated adequate shelf-life stability through formulation optimization efforts. They also demonstrated maintaining physical integrity and successful removal of the device up to 6 months after insertion *in vivo*, allowing for analysis of residual drug recovered from the device.

Some challenges that were observed with this device *in vitro* and *in vivo* with TAF included: degradation of TAF inside the implant owing to TAF sensitivity to water/phosphate-buffered saline (PBS) in the *in vitro* experimental set up and the increasing acidity of the implant microenvironment; if this level of drug degradation would be similar *in vivo* and acceptable from a regulatory perspective; the need to identify all degradation products *in vivo*; an observed discoloration of the implant over time; and, some local reactivity of TAF *in vivo*.

They also reported a tunable release rate of TAF free base that ranged from 0.20 to 1.0 mg/day with implants produced from research grade PCL. Primary variables for controlling release included implant wall thickness, the oil excipient (e.g., sesame vs. castor oil), and the physical properties of the PCL^30^ (e.g., molecular weight, % crystallinity). They were also successful at identifying formulation additives that helped with the stabilization of TAF in aqueous environments (e.g., control of pH with the inclusion of sodium citrate in the drug formulation).^31^ The advances made in terms of the product optimization allowed the group to select three candidate formulations for delivery of TAF in a dog model.

#### *In vivo* animal studies

RTI conducted PK and local safety studies in three animal models: rabbit, dog, and NHP. The dog studies were carried out with the three optimized formulations, which were engineered for three different release rates through tube wall thickness, PCL molecular weight, and excipient selection. Results of the 6-month dog study were produced for three different delivery doses per day: dose no. 1, 0.16 mg/day; dose no. 2, 0.26 mg/day; dose no. 3, 0.36 mg/day.^32^ Two of the three dogs in the placebo arms of doses no. 1 and no. 2 and all three dogs in the placebo arm of dose no. 3 completed the 182-day study, as did two of the three dogs in the active arm of dose no. 1. None of the active arm animals for dose no. 2 completed the study, and one of the three dogs in the active arm of dose 3 completed the study.^32^

Important PK and safety findings in this study were as follows: (1) stable and low plasma levels of TAF and TFV were observed throughout study duration; sustained TFV-DP levels measured at >200 fmol/10^6^ PBMC for up to 6 months with rapid drop in PBMC concentrations within 2 weeks of implant removal; (2) site lesions/abscesses associated with drug dumping and poor device integrity early in the study (formulation 2); and long-term, chronic exposure (formulations 1 and 3). No animals were killed prematurely and all animals were cleared to return to stock as lesions were reversible within 2 weeks of implant removal.^31^

A second *in vivo* study was conducted with these devices in rhesus pigtail macaques.

A quantitative summary of safety findings in this NHP study is provided in Table 3.^33^ Key PK and safety findings from this NHP study included the following: (1) low sustained TFV exposure in plasma (i.e., below limit of quantitation [BLOQ]); however, high sustained levels of TFV-DP in PBMC were observed; (2) all the high-dose animals completed the PK study, showing mild-to-moderate skin irritation/toxicity with long-term use. Hematoxylin and eosin (H&E) staining of tissue surrounding the low- and medium-dose implants revealed moderate to marked deep dermal inflammation.

**Table 3.** Quantitative Summary of Safety Findings in RTI Nonhuman Primate Study

Implant formulation/dose	Duration (weeks)	Grade 0 local reactivity^a^	Grades 1–2 local reactivity^a^	Grades 3–4 local reactivity^a^
No. 1/0.16 mg per day^b^	8	7/21 (33%)	13/21 (62%)	1/21 (5%)
No. 2/0.35 mg per day^c^	11–12	14/62 (23%)	39/62 (63%)	9/62 (14%)
No. 3/0.70 mg per day	20	57/114 (50%)	46/114 (40%)	11/114 (10%)

^a^ Denominators listed in these columns are the total number of cage side observations per implant dose group.

^b^ Implant failure (*n* = 1, week 5).

^c^ Implant came out of animal (*n* = 1, week 5).

### Intarcia Therapeutics Inc.: titanium osmotic mini-pump implant

**FIG. 2.**
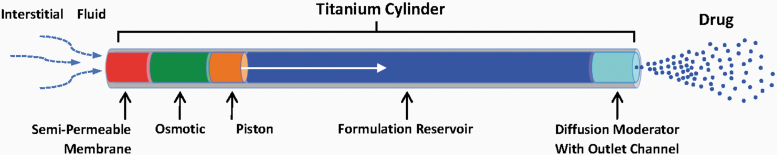
The osmotic mini-pump is placed in the subdermal space. Interstitial fluid flows consistently and predictably through the semipermeable membrane into the osmotic engine compartment that contains NaCl tablets. Mixing of the salt with fluid causes expansion of the osmotic engine compartment that pushes a piston resulting in formulated drug being expelled through the diffusion moderator into the SC space where drug is absorbed into the systemic circulation. NaCl, sodium chloride; SC, subcutaneous.

This technology (originally developed by the ALZA Corporation and is based on the DUROS delivery technology) is a sterile, nondegradable implant designed to achieve zero-order release kinetics of drugs for up to 1 year.^24^ The body of the device is a cylindrical titanium alloy capped at one end by a water permeable membrane, and at the other end by a control diffusion modulator (DM), from which drug is expelled (Fig. 2). The mini-pump has an engine compartment that contains sodium chloride (NaCl) to create an osmotic gradient across the membrane. Through the processes of osmosis, fluid is absorbed from the outside environment through the membrane and into this osmotic engine compartment. The increasing volume of water entering the mini-pump expands the osmotic engine compartment and pushes a piston that drives formulated drug in the reservoir through an open channel in the DM. There is enough NaCl content in the mini-pump's engine compartment to maintain a saturated salt solution throughout the in-use period, thus maintaining a constant pressure gradient and steady release of drug into the SC space. When drug has been exhausted from the device, it must be removed and replaced by a new one to maintain necessary drug exposure levels.

The Good Laboratory Practices (GLP) safety and PK studies performed by Intarcia delivered TAF in an aqueous vehicle by a continuous infusion through a SC cannula and, thus, were relevant to the overall preclinical assessment of SC delivery of TAF for HIV prevention by any LA SC delivery technology. Of importance, these specific studies were informed by nonclinical PK and pilot safety work conducted by Intarcia and other data sources.^20,21,34^ For example, an Intarcia pilot study in rats in which the hemifumarate salt of TAF was administered by continuous SC infusion for 14 days led to no signs of systemic toxicity with very slight edema local to the administration site being observed in two of four animals treated at 1.08 mg/kg/day, as compared with similar observations in just one of four animals in the vehicle group.

Based on the TFV exposure levels measured in Intarcia's 14-day pilot tolerability study at 1.08 mg/kg/day (TFV AUC_24h_ at steady state = 0.237 μg × h/mL), the high dose of 1 mg/kg/day was selected for the GLP toxicology study and was expected to achieve exposures below that measured at the no adverse effect level (NOAEL) in rats after 28 days of daily oral exposure of TAF (6.25 mg/kg/day; day 28 TFV AUC_(0–*t*)_ = 0.340 μg × h/mL).

Dose levels for dog infusion studies were similarly informed by earlier studies conducted by Intarcia and Gilead. The high dose in the Intarcia GLP dog 28-day toxicity study (833 μg/kg/day) was expected to produce similar systemic exposure to that observed at the oral dose NOAEL in the 9-month oral dog toxicity study reported by Gilead.^34^ In the Gilead study, a number of findings were reported at doses 3 × the NOAEL level, and included the following: body weight loss, minimal renal toxicity, slightly prolonged PR intervals in the heart, pulmonary changes, and minimal bone loss. Therefore, it was expected that the highest dose in Intarcia's planned SC infusion GLP toxicity study may produce local effects but that there would be no systemic findings, and that the lowest dose would not generate local or systemic findings.

#### Rat toxicity results: Intarcia 28-day infusion study

The design of this rat study^35^ involved the administration of TAF hemifumarate to rats by continuous SC infusion for 28 days and resulted in findings at the infusion site including: (1) exacerbation of infusion site lesions in males at ≥30 μg/kg/day, (2) macroscopic finding of a mass (all males at 1,000 μg/kg/day), (3) dose-related increased incidence and severity of mixed cell inflammation (most males, ≥30 μg/kg/day), (4) increased incidence and/or severity of fibrosis and mononuclear cell inflammation (most males, ≥300 μg/kg/day), (5) increased incidence and severity of necrosis (all males at 1,000 μg/kg/day), and (6) presence of Gram+cocci bacteria within the infusion site (some males and females in both control and treatment groups).

The presence of bacteria within the infusion sites was considered secondary to skin ulceration (opportunistic infection) and unrelated to the administration of hemifumarate TAF. At the end of the recovery phase (day 57), macroscopic and microscopic findings noted at the infusion site were of reduced incidence and/or severity compared with the treatment phase. This suggests ongoing resolution of TAF-related exacerbation of infusion site lesions. Local and systemic NOAELs were considered to be 1,000 μg/kg/day. Based on these NOAELs, the estimated clinical margin for local inflammation was two- to three-fold (total dose at 1,000 μg/kg/day was 300 and 500 μg/day in females and males, respectively), and the estimated clinical margin for systemic exposure was 87.5-fold for TFV, but only 3.3-fold for intracellular TFV-DP. TAF concentrations were below the limit of quantification (<0.01 ng/mL) in all samples from this study.

#### Beagle dog toxicity results: Intarcia 28-day infusion study

This preclinical *in vivo* study was conducted in beagle dogs.^35^ Preterminal killing of several animals, including two controls and all animals administered 0.833 mg/kg/day, was conducted because of swelling at the infusion site, discharge at the infusion site, and deteriorating conditions of the animals. Clinical observations noted at ≥0.025 mg/kg/day were associated with inflammation at the infusion site, supported by hematology, coagulation, and clinical chemistry changes confirming an inflammatory process. Macroscopic and microscopic observations confirmed mononuclear cell inflammation, mixed cell inflammation, and necrosis at the infusion site.

Because of the severity of the observations noted, a NOAEL for local findings could not be established for this study, essentially providing no clinical safety margin for local inflammation, given that the lowest dose of TAF tested in this study was 0.025 mg/kg/day or 0.18 mg/day total dose. It should be noted that results from this study were confounded by: (1) the need to treat the same animals during the study with anti-inflammatory, antibacterial, and/or opioid agents to enable the animals to complete the study; and (2) the use of the continuous SC infusion set up led to local tolerability issues that were observed in vehicle and treated animals. However, the local toxicities were exacerbated by TAF administration.

There were no TAF-related effects that resulted from systemic exposure to TAF at the highest remaining dose group tested. The NOAEL for the systemic exposure to TAF, excluding any infusion site-related findings, was considered to be 0.25 mg/kg/day, which provided estimated clinical exposure margins 1,523-fold for TAF, 65.5-fold for TFV, and 93-fold for intracellular TFV-DP.

The development efforts of these three foundation-funded partners all used different technologies to achieve LA delivery of TAF: injection, degradable implant, and nondegradable implant. In addition to the work of these partners, other groups who were working independently of the foundation to develop LA TAF delivery products with other sources of funding were also invited to the 2020 LA TAF Meeting. Those groups, their technologies, and their study findings are summarized in the following sections.

### NW (SLAP-HIV grant; funded by NIH/DAIDS): nondegradable reservoir implant

The SLAP-HIV program is an NIH/DAIDS-funded grant at NW, focused on the development of LA HIV prevention products. The SLAP-HIV group had been involved in the development of a dry pellet TAF reservoir implant comprising a polyurethane (PU) tube, originally sealed using an adhesive but eventually sealed with heat welding. Differing configurations and materials of these implants leads to different *in vitro* and *in vivo* release rates. This device is designed with 150–170 μm thin PU tubing that is loaded with TAF hemifumarate plus small amounts of NaCl and magnesium stearate. This design functions as a “physical capsule” with the tube wall composition, wall thickness, and overall size of the implant regulating the rate of drug release. There were two “generations” of implants (A and B) used in the following rabbit and NHP studies. Technical differences and *in vitro* performance data were provided in Su *et al.*^25^

Multiple configurations of the generation A device were evaluated in New Zealand white rabbits and the rhesus NHP model for PK and safety (primarily histopathology) and the results of these studies were published.^25^ This rabbit study design involved surgical implantation of devices in 12 rabbits behind the neck (6 placebo, 6 treated), 2 of each were killed at 28 days and the remainder were killed at 3 months for histopathology assessments. Blood samples were drawn weekly for 3 months for analysis of plasma and PBMC PK, and bimonthly mucosal samples were obtained by vaginal and rectal biopsies to measure tissue levels.

At day 28 the treated rabbits demonstrated focal granulomatous inflammation around the area of the implants. By 90 days, liquefactive and coagulative necrosis was observed in the treated animals at the sites of implantation. These findings were not observed in rabbits with placebo devices. All rabbits containing TAF-loaded plug-sealed implants had inflammation around the implant site, often closest to one or both polar regions of the implant. Animals with “capped” implants, which were prone to sporadic leaking, showed necrosis and perivascular and perineural inflammation with cuffs of lymphocytes and macrophages surrounding the implant sites, and outside the implant and tissue capsules. A second rabbit study was conducted that involved some “fixes” to the original study (i.e., placement of implants to avoid self-removal, reduced drug delivery, heat sealed ends, barium pellets to find implants lost in animals, and institution of a histopathology scoring system). The study involved dose ranging of TAF that resulted in TFV-DP levels ranging from 68 to 391 fmol/10^6^ PBMC**.** At 28 days postimplantation, histopathology findings were similar to those seen in the first rabbit study: multicell infiltration inflammation and necrosis at the sites of TAF implants, and little to no findings with placebo implants.

Two generations of implants (A and B) were evaluated for PK and safety in the NHP model with a 12-week study.^25^ Although there was a slightly higher tissue response associated with the placebo implant in the NHP relative to the rabbit model, meaningful adverse histopathology findings were associated with TAF implants in the NHPs. In the cases of many animals, fibrosis, hemorrhagic abscesses, and severe granulomatosis were observed with TAF implants. In some instances, implants were lost (via extrusion from the animals) or fell apart *in vivo*. However, some NHPs had only a minimal increase in adverse response to the TAF implant relative to the placebo. This was illustrated utilizing a new semiquantitative histopathology scoring system to evaluate histopathologic responses to the placebo and TAF implants that were placed in the same animal.^25^

For example, in one animal the placebo implant had a thin capsule and mild pericapsular infiltrates of lymphocytes and plasma cells, but the periphery of the thin capsule was associated with multifocal aggregates of lymphocytes, plasma cells, edema, and hemorrhage giving a score of 16. The TAF implant in this animal had a thick capsule filled with proteinaceous fluid, heterophils, plasma cells, macrophages as well as extensive fibrosis and lymphoplasmacytic inflammation extending into adjacent tissues generating a score of 24. The adverse histopathology score was greater in the TAF implant relative to placebo in all animals. A subsequent pilot study where the TAF implants were inserted utilizing a trocar likewise resulted in adverse histopathological results.

In light of the findings of these animal studies, the SLAP-HIV program abandoned further development of TAF implants and switched to the use of CAB.

### Houston Methodist Research Institute: nondegradable transcutaneously refillable nanofluidic implant

**FIG. 3.**
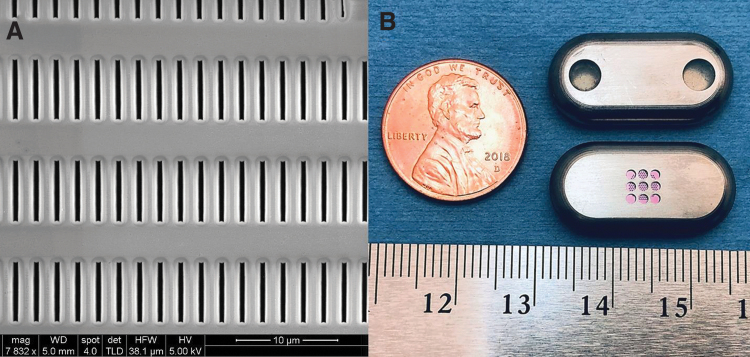
**(A)** Scanning electron microscopy image of the surface of the nanofluidic membrane, showing the inlet of the slit-nanochannels and the dense channel array. **(B)** Nanofluidic implant displaying the two loading and venting ports with self-sealing septa (*top*) and the nanochannel membrane (*pink*) assembled within the titanium drug reservoir (*bottom*).

This is an implantable nanofluidic technology that regulates diffusive drug release using silicon nanochannel membranes. The silicon membranes are nanofabricated, adopting technologies from the semiconductor industry^36^ achieving drug delivery through *slit*-nanochannels etched perpendicularly to the membrane surface. These nanochannels are densely stacked in a titanium reservoir implant as regular square arrays coated with silicon carbide for long-term bioinertness and biocompatibility^37^ (Fig. 3A).

The nanochannel membrane is assembled within a titanium reservoir implant and serves as the rate-limiting component for drug release^26^ (Fig. 3B). Unlike other implantable delivery system, the technology is “drug and formulation agnostic”^38^ and can be used with both liquid and solid formulations of drugs irrespective of their molecular properties (charge, hydrophobicity, hydrophilicity, molecular weight, and structure).^39^ In the case of TAF, initial drug loading of the device is achieved by packing drug powder into the implant.^40^ Once the implant reservoir is depleted, drug can be refilled transcutaneously through palpable “refill” ports, which avoids repeated surgical insertion and retrieval procedures. Implant refilling is performed using a loading and a venting needle and can be operated with both liquid and solid drug formulations. The drug is injected through the loading port and the venting needle allows for flushing the reservoir and device refill.^26^

Once the nanofluidic implant is inserted subcutaneously, the drug release is initiated by the influx of interstitial fluids penetrating into the device by capillary wetting of the membrane and solubilization of a portion of the drug powder formulation. Then solubilized drug molecules diffuse across the membrane into the surrounding tissues. This establishes a continuous mechanism of solubilization and release that allows for high drug loading efficiency and promotes formulation stability, long term. Sustained and constant rates of drug release are achieved through electrostatic and steric interactions between drug molecules crossing the nanochannels and confining channel walls.^41,42^ No pumps or valve and actuators are needed for drug elution. Rate of release is controlled by the nanochannel membrane configuration^39^ (i.e., nanochannel size and number).

Although solid drug loading limits drug stability issues, TAF presents poor stability in the presence of water. One strategy to achieve enhanced stability of TAF released from the nanofluidic implant is with the use of a buffering agent for pH control in the range of 5.0–5.5.^43^ Because of their greater solubility than TAF, common buffers such as citrate buffer are not able to sustain the pH in the desired range long term, as they are depleted by diffusion out of the implant reservoir at a much higher rate than TAF. To address this, a viable approach is using a low solubility buffer such as urocanic acid, which is released from the implant at a similar or slower rate than TAF. By leveraging the buffering properties of urocanic acid, this group achieved extended stability of TAF released from the nanofluidic device for over 9 months.^43^ The additional formulation volume of urocanic acid is smaller than the volume gained by removing the fumarate group from TAF. In other words, the TAF-urocanic acid formulation enables the loading of 500 mg of formulation, which establishes a longer duration of release for this device.

The primary focus from this group was their NHP efficacy study, which was conducted with the support of Gilead and NIH.^44^ The study involved 14 rhesus macaques (7 females and 7 males). Eight animals (four females and four males) received the SC TAF implant (PrEP group) in the dorsum. The control group (three females and three males) received a vehicle (PBS-loaded implant). An interesting element of this study is that rectal viral challenge was initiated only after the animals had reached levels of TFV-DP believed to be consistent with protection (TFV-DP preventive level considered to be 100 fmol/10^6^ PBMCs).

The dose of TAF achieved in the animals with this device was ∼1.4 mg/day, which resulted in sustained levels of TFV-DP in PBMC of ∼500 fmol/10^6^ PBMCs, well above the anticipated level required for protection against HIV infection. After 10 weekly rectal exposures, two PrEP animals remained uninfected. An additional animal in the PrEP group displayed transient infection with undetectable viral load 5 weeks after removal of the implant and cessation of TAF administration. The control cohort was 3.04 times more likely to be infected after the fourth rectal challenge dose.^44^

These results are similar to those obtained with TDF alone in the human Partners PrEP trial, which possibly explains the lower efficacy observed in this study with TAF monotherapy. One of the hypotheses offered to explain the result of this NHP study was that SC delivery of TAF may not achieve sufficient drug concentration in the rectum. An additional hypothesis is that a drug combination such as FTC/TAF would be required for enhanced synergistic efficacy in a rectal challenge model. In this context, this group is exploring the use of the nanofluidic implant for the sustained long-term delivery of different ARV including FTC, CAB,^45^ and islatravir. Although this study was not a comprehensive toxicity evaluation of this technology and TAF delivery, blinded pathology assessment of tissues surrounding the TAF implant (4 months of implant use) by three independent clinical laboratories displayed a normal foreign-body response with no inflammatory cell infiltration.^43^ Specifically, histopathological examination was performed according to the scoring system used by the SLAP-HIV group.^25^ Tissue response to the nanofluidic TAF implants was qualified as “slight reaction.” Of note, these results are in significant contrast with other results obtained with polymeric implants (e.g., Su *et al.*^25^), for which “severe” tissue response was observed despite an order of magnitude lower TAF release rate.

### Oak Crest: silicone reservoir implant

This group developed a silicone tube reservoir TAF implant based on a cylindrical silicone scaffold that achieves linear drug release through a controlled number and size of specific delivery channels in the impermeable sheath as well as an outer PVA membrane that covers the channels. The implant is packed with solid TAF powder, or microtablets, and controlled drug release is achieved through the delivery channels as *in vivo* fluids enter the device and dissolve the drug. The implant scaffold comprises medical grade, platinum catalyzed silicone tubing with an inter diameter of 1.5–2.0 mm and an outer diameter of 1.9–2.4 mm. Additional details of this device were provided in a publication from this group.^20^ They observed better drug release control with the free base form of TAF versus the hemifumarate and release was linear *in vitro* for 6 months.

Although the first *in vivo* PK study was conducted in dogs for 40 days, they later evaluated a variety of implant prototypes in dogs, mice, and sheep over multiple studies^46^ primarily for PK evaluation. The team did not have concerning toxicity findings in any species with doses ≤1.0 mg/day. The question of TAF metabolites distribution in the dermal tissues adjacent to the implant possibly being responsible for the safety findings found in other studies was raised, particularly in terms of dose relative to animal size. It was noted that there is difficulty in performing scaling between different body weights with respect to local dermal concentrations of TFV and TFV-DP as evidenced by the observation of significant differences between sheep and mice. Results indicate that there is no equivalent local buildup of drug (i.e., exposure) for implants of the same release rate.

In addition, it was observed in these studies that there were high levels of TFV and TFV-DP in dermal tissues close to the implant with little or no local tolerability issues. An important difference between these mouse and sheep PK studies and that of some of the other groups is that this device was delivering the free-base form of TAF, whereas some other studies with more significant toxicological findings were delivering TAF hemifumarate and used implants of different materials and manufacturing processes. Consequently, this development team believes it is possible that TAF or one of its metabolites, possibly in conjunction with the fumaric acid, are responsible for the observed toxicity in those models demonstrating more significant findings. Addressing these hypotheses would require additional preclinical studies. The questions of safety and PKs of the Oak Crest TAF free-base implants will be addressed to some degree in a planned trial in humans (the protocol for this trial, CAPRISA 018, is available at CAPRISA.org (file:///D:/Downloads/CAPRISA%20018_Study%20protocol %20V2.0_12%20Aug%202019.pdf).

**FIG. 4.**
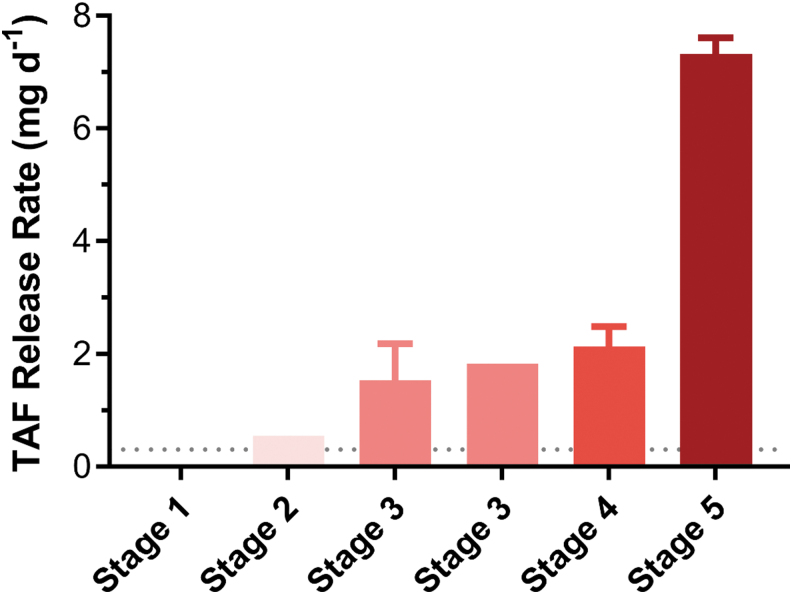
Stage 1, scab, slight erythema; stage 2, slight swelling at dose site, scab, slight edema; stage 3, mild-to-moderate edema, scab, swelling, ocular discharge, emesis; stage 4, macroscopic descriptions of swelling and/or firmness in the interscapular implant sites, no expressible fluid; stage 5, slight swelling, emesis, red-tinged material at dose site and yellow discharge; mild edema, mild erythema.

The Oak Crest group also reported some safety findings in dose escalation studies in dogs for their implant device. As noted previously, there were no safety observations made with doses <1.0 mg/day, which is encouraging. However higher doses did lead to safety observations, as given in Figure 4.^47^

#### Other results and data comparisons

Of interest, different products were evaluated for PK in the same animal models. Results from the use of common models (rabbit and dog) that were provided at the meeting indicated that different products have somewhat differing results in terms of PK in the same animal model, which was not unexpected given the differences in TAF used (hemifumarate vs. freebase) and the release differences observed with each delivery device.

#### Preclinical assessments of TAF efficacy

The most relevant preclinical efficacy data comes from the group at Houston Methodist. Their refillable SC implant that delivers TAF with apparent zero-order release kinetics was used in a rectal challenge study in rhesus macaques in partnership with Gilead.^44^ The dose estimated to be delivered from this device in this study was 2.0 mg/day. The PK clearly demonstrates sustained steady-state plasma concentrations of TFV and sustained levels of TFV-DP in PBMCs at ∼500 fmol/million cells. An interesting part of the study design was that the challenges were not started until the TFV-DP levels exceeded what is thought to be an effective level (∼100 fmol/million cells). Despite the apparently appropriate PK profiles and high levels of TFV-DP, infection was delayed but the TAF was not fully protective (∼70% efficacy). The control animals were all infected after four challenges.

Gerardo Garcia-Lerma *et al.* [Centers for Disease Control and Prevention (CDC)] generated NHP challenges data for TAF and FTC/TAF dosed orally. In their original study, rhesus macaques were given a high dose of TAF and challenged rectally with simian HIV (SHIV) 3 days later. This study showed no protection.^7^ More recently they tested the efficacy of oral TAF and F/TAF in pigtail and rhesus macaques using rectal^8^ and vaginal^9^ challenges. In these more recent experiments, animals were dosed 24 h prior and 2 h postchallenge at 1.5 mg/kg. TFV-DP levels of >100 fmol/million PBMCs were generally achieved. Five of nine pigtail macaques treated with TAF and exposed to SHIV vaginally became infected during the study (15 challenges). Two of these animals, however, did not achieve protective levels of TFV-DP for reasons that are not clear. Excluding these animals, 4 of 7 of treated animals were protected, but 20 of 21 controls became infected (58%–73% efficacy). A rectal challenge study in which rhesus macaques received FTC/TAF (TAF dose 1.5 mg/kg) orally 24 h before and 2 h after SHIV challenge showed complete protection. This same combination of drugs tested in the pigtail macaque vaginal challenge model conferred 91%–100% efficacy. A summary of these data is given in Table 4. These results suggest that TAF in combination with another drug may be necessary to prevent infection (e.g., FTC/TAF), which would be consistent with the reduced efficacy reported with TAF alone in the Methodist Hospital results, summarized earlier.

**Table 4.** Summary Macaque Efficacy Data with Oral Tenofovir Alafenamide (TAF) and FTC/TAF

	Rectal challenge (rhesus macaques)	Vaginal challenge (pigtail macaques)
TAF	Supra therapeutic dose 13.7 mg/kg	Clinical dose (1.5 mg/kg)
	∼1,600 fmol/10^6^ PBMC	∼300 fmol/10^6^ PBMC
	∼1,000 fmol/10^6^ rectal lymphocytes	∼5 fmol/mg of vaginal tissue
	No protection	58%–73% efficacy
FTC/TAF	Clinical dose (1.5 mg/kg)	Clinical dose (1.5 mg/kg)
	100% efficacy	91%–100% efficacy

PBMC, peripheral blood mononuclear cell.

## Conclusions

The studies and efforts summarized at the foundation meeting on the development of LA products for the delivery of TAF for HIV prevention led to a number of important conclusions. Despite the fact that multiple technologies demonstrated that LA delivery of TAF in the form of SC implants was viable, there was also clear demonstration in nonclinical animal models that SC delivery of TAF could lead to safety and/or tolerability issues during clinical development. There are several possible explanations for the observed toxicity, including: differences related to delivery of TAF hemifumarate versus free base; rate of local drug release; the potential impact of TAF metabolite release or production in the local tissue; the local release of excipients; and combination effects because of local deposits of excipients and metabolites.

Furthermore, it was also shown that TAF alone may not be adequate to achieve an appropriate level of protection from HIV infection in comparison with other products currently in development. Consequently, it was concluded that additional investment in LA TAF product development efforts was not appropriate. However, the performance of the delivery technologies suggested that LA delivery of other drugs with better safety and efficacy profiles could be viable going forward.
